# Efficient surface polishing using burst and biburst mode ultrafast laser irradiation†

**DOI:** 10.1039/d2ra05208c

**Published:** 2023-01-25

**Authors:** Mantas Gaidys, Andrius Žemaitis, Paulius Gečys, Mindaugas Gedvilas

**Affiliations:** a Department of Laser Technologies (LTS), Center for Physical Sciences and Technology (FTMC) Savanorių Ave. 231 LT-02300 Vilnius Lithuania mantas.gaidys@ftmc.lt

## Abstract

The use of laser irradiation for micromachining is widely applicable and has many benefits. One of the main uses is that it is possible to mill and polish the sample using the same laser system. State-of-the-art laser systems with high average optical power and burst regimes are widely used in technology. The main advantages of burst regimes are the closer fluence values to optimal fluences and residual heat reusage for subsequent pulses. In this study, the influence of MHz burst, GHz burst, and bibursts was investigated for significant surface polishing of copper and stainless-steel samples. Z-scan experiments were performed to determine the optimal number of sub-pulses inside the burst for the lowest surface roughness.

## Abstract 2

The use of laser irradiation for micromachining is widely applicable and has many benefits. Using correct parameters in burst and biburst modes 0.1 μm surface roughness is achievable with laser polishing.

## Graphical abstract

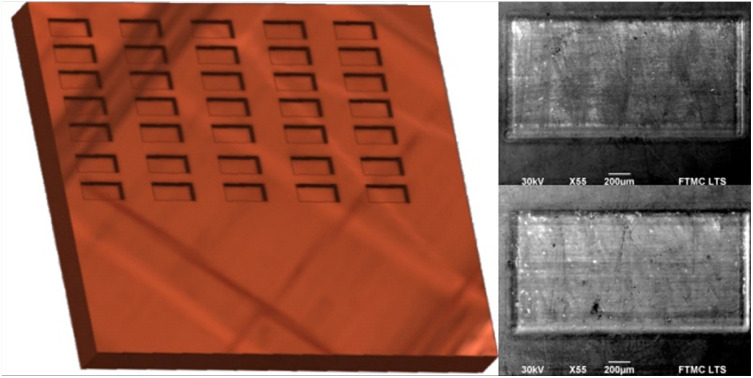

## Introduction

Many products used in various industries are significantly influenced by texture and surface quality.^1^ Laser micromachining is an appealing method because of the abundance of applications: from cutting^2–5^ and drilling^6–8^ of various materials to forming two and a half dimensional (2.5D) structures on various surfaces.^9,10^ Because sub-micrometer surface roughness is now desired, laser polishing is a prospective polishing method. Laser polishing allows for high precision, good finish quality, a vast range of possible materials, reaching delicate places, polishing complex forms,^11,12^ and the ability to micromachine and polish samples using the same laser system.

However, optimal laser processing parameters, such as pulse repetition rate, pulse energy, and pulse overlap, have to be chosen correctly to achieve the best results, which are tedious and time-consuming. Lasers and systems designed for laser micro/nano-fabrication are improved daily. It is now possible to have systems that use MHz and GHz repetition rates with femtosecond laser pulses and high average optical power using burst and biburst modes.^13^ These modes allow a significant increase in ablation efficiency^14,15^ and significantly improve the ablated surface roughness [15] if the laser processing parameters are optimized correctly. Laser bursts also provide faster processing owing to burst energy division into pulse energies closer to optimal fluences, resulting in the exploitation of more average laser power.^16^ Moreover, because of the short time between burst pulses, any residual heat from the previous pulse is reused.^17^

In this study, a state-of-the-art laser working in burst and biburst modes was used for copper and stainless-steel milling and polishing. The number of sub-pulses in MHz and GHz bursts and biburst were controlled. The surface roughness measured by the stylus profiler was investigated as a parameter for defining polishing quality. Different numbers of pulses in burst and biburst regimes resulted in different surface roughness of laser-milled areas in copper and stainless steel. The optimal laser processing parameters were found to achieve the best laser polishing quality with a mirror-like finish.

## Materials and methods

### Experimental setup

**Fig. 1:**
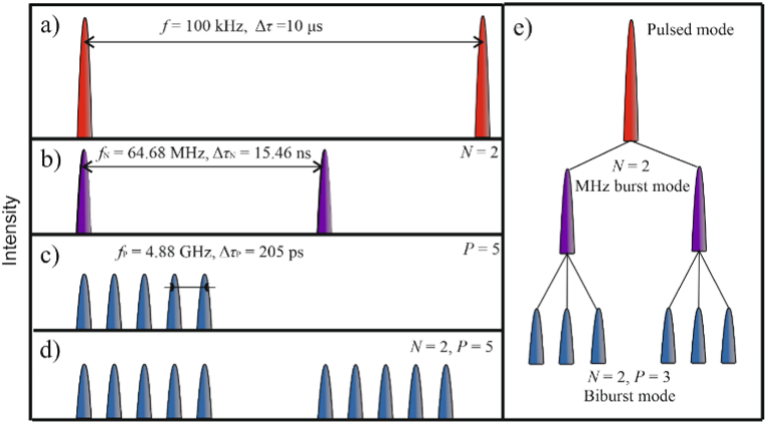
Schematic representation of different regimes of the laser irradiation. (a) Standard pulsed regime with a pulse repetition rate of *f* = 100 kHz, (b) MHz burst regime with a pulse repetition rate of *f*_N_ = 64.68 MHz, and the single pulse split into *N* = 2 pulses. The time between burst pulses Δτ_N_ = 15.46 ns, (c) GHz burst regime with burst pulse repetition rate of *f*_P_ = 4.88 GHz, and the single pulse split into *P* = 5 pulses. The time between burst pulses Δ*τ*_P_ = 205 ps (d) biburst regime, where the single pulse is split into *N* = 2 pulse MHz burst that are subsequently split into *P* = 5 pulse GHz burst, and (e) different visualization of pulsed regimes division into MHz and biburst modes.

A state-of-the-art solid-state laser (Pharos, Light Conversion) with an average optical power of 7.3 W at a wavelength of 1030 nm was used for the experiments. The pulse duration was *τ* = 210 fs, and the repetition rate was *f* = 100 kHz (Fig. 1a). The laser system can operate in a pulsed regime and several burst regimes. Three different burst regimes exist: MHz burst, GHz burst, and biburst. While using the MHz regime, a single laser pulse could be divided into multiple pulses with a repetition rate of *f*_N_ = 64.68 MHz, corresponding to Δ*τ*_N_ = 15.46 ns between pulses (Fig. 1b). During the experiment, the burst was divided from *N* = 1 to *N* = 9. While using the GHz burst regime, a single pulse could be divided into numerous pulses with a repetition rate of *f*_P_ = 4.88 GHz, corresponding to Δ*τ*_P_ = 205 ps between pulses (Fig. 1c). During the experiment, the pulse was divided from *P* = 2 to *P* = 25. Finally, using the biburst regime, a single pulse could be divided into several MHz pulses, and each of the MHz pulses could be further divided into several GHz pulses (Fig. 1d). Another visualization of pulse division into subpulses is presented (Fig. 1e).

**Fig. 2:**
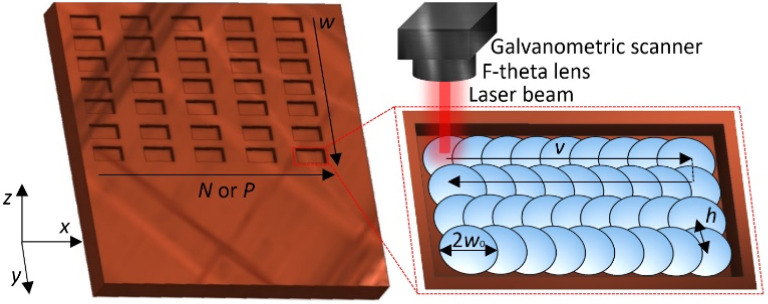
Design of laser milling and polishing experiment. Rectangle cavities were ablated in a copper and a stainless-steel sample. Beam size was changed in columns by changing the position of the sample. *N* or *P* were changed in matrix lines. Here, *v* is the scanning speed and was kept constant at 333 mm s^−1^, *h* is the hatch and was 10 μm, *w*_0_ is the beam radius and was changed from 21 μm to 95 μm. The dashed line represents the mirror jump of galvanometric scanners.

The laser beam was focused on using a 100 mm focal distance F-theta lens, and the beam was controlled in the *x* and *y* directions by applying a galvanometric scanner (Intelliscan 14, Scanlab). The beam radii values were needed in various sample *z* positions during the experiment and were measured using a well-known *D*-squared method. The beam radius in the focal plane was equal to 21 μm. The rectangular-shaped cavities with a top dimension of 2 mm × 1 mm were ablated in the copper and stainless-steel samples (Fig. 2). The distance between the scanning lines was kept constant at 10 μm, and the scanning speed was set to 333 mm s^−1^. The sample position was changed in the *z*-direction to obtain various fluence values. Each rectangle was scanned numerous times to achieve a depth that could be easily measured using a stylus profiler (Dektak 150+, Veeco). During the MHz and GHz burst experiments, the number of pulses in bursts was changed from *N* = 2 to *N* = 9 and from *P* = 2 to *P* = 25, respectively. During the biburst experiment, every N and P combination was tested.

### Beam characteristics

To change the laser fluence, the sample *z* position was changed. The beam radius increased when the sample was moved in either the positive or negative *z*-direction. A well-known *D*-squared method was used to calculate the different beam radii values.^18^ During this method, a sample is damaged using laser irradiation at various average laser powers, and the radius of the damage is measured using an optical microscope (BX51, Olympus). A graph of the squared radius of the irradiated damage dependence on fluence is drawn. Using eqn (1) of linear fit on the graph, we obtain the beam radius from the slope at the current sample *z* position. This experiment was repeated for all *z* values, which were 0.2, 0.4, 1.0, 1.4, 1.9, 2.4, 2.7, 3.1, 3.8, and 4.9 mm in both directions. We have1
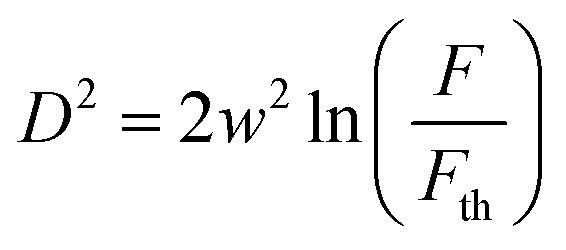
where *D* denotes the diameter of the laser irradiation damage of the sample, *w* denotes the beam radius at a given *z* position, *F* denotes the fluence, and *F*_th_ denotes the ablation threshold.

**Fig. 3:**
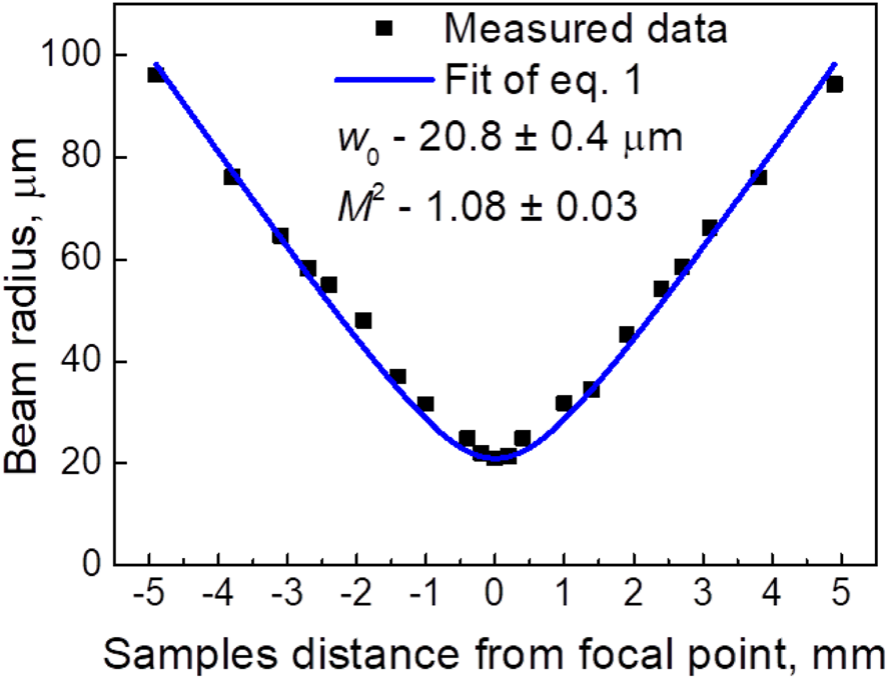
Laser beam radii at different sample *z* positions. Black squares represent experimental data, and the blue line represents the fit of equation. The received parameters were beam waist *w*_0_ (20.8 ± 0.4 μm) and beam quality parameter *M*^2^ (1.08 ± 0.03).

Measured beam radii values are shown in (Fig. 3). We obtain2
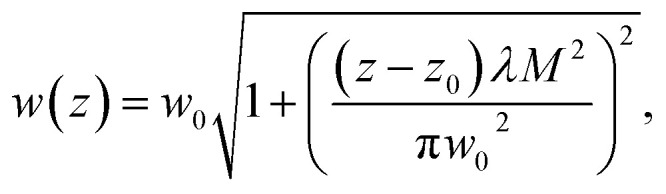
where *w*_0_ denotes the beam waist, *z*_0_ denotes the sample position at the beam's waist, *z* denotes the sample current position, and *λ* denotes the laser wavelength, which was 1030 nm, and *M*^2^ denotes the beam quality parameter.

### Sample characteristics

For the experiments, 5 mm × 50 mm × 50 mm copper (CW004A, Ekstremalė) and stainless-steel (1.4301, Ekstremalė) samples were used with a surface roughness *R*_a_ of 0.1 μm and 0.5 μm, respectively. The copper purity was 99%. The ablated surface roughness *R*_a_ was measured using a stylus profiler (Dektak 150, Veeco). A scanning electron microscope (JSM-6490LV, JEOL) was used to visualize the samples.

## Results and discussion

Laser polishing is a technique in laser micromachining that entails melting a thin layer of a metal surface that is free of fractures and other defects. Using a pulsed laser system, this is done by micro polishing, where the beam is moved along the target's surface to melt the peaks into the valleys and, consequently, gain a more even surface. The molten material is already re-solidified when the subsequent laser pulse hits the surface and creates a new molten pool.^19^ Shallow surface melting is the primary cause of this phenomenon. The shallow surface melting zone is created by capillary pressure and liquid curvature caused by the shallow melting of micro asperities, which finally fill the valleys of the metal surface with molten metal.^20^ Laser ablation is also very useful for laser polishing. In real-world applications, during the free form ablation process, some ablated debris did not leave the area and instead was deposited back onto the irradiated surface. How big and common those debris are is a probabilistic process that depends on the laser parameters and the geometry of the ablated region. Some larger debris may not be removed with laser surface melting compared to laser ablation. The intensity of the laser beam must be chosen based on the pulse duration and the type of polished material. Consequently, one of the most important parameters for effective laser polishing is laser fluence, which is easily controllable using laser bursts. Even slight changes in fluence can result in a wide range of surface quality finishes. Another major factor is the temperature of the surface of the sample. In the case of stainless-steel, studies have revealed that a surface temperature reaching 600 °C before the next pulse significantly affects surface roughness.^21^ However, the accumulated heat can also be used as an advantage when using various burst modes. According to many studies, the use of burst modes with fluences lower than the ablation threshold fluence results in a thin melt layer that enhances surface quality.^22–24^

**Fig. 4:**
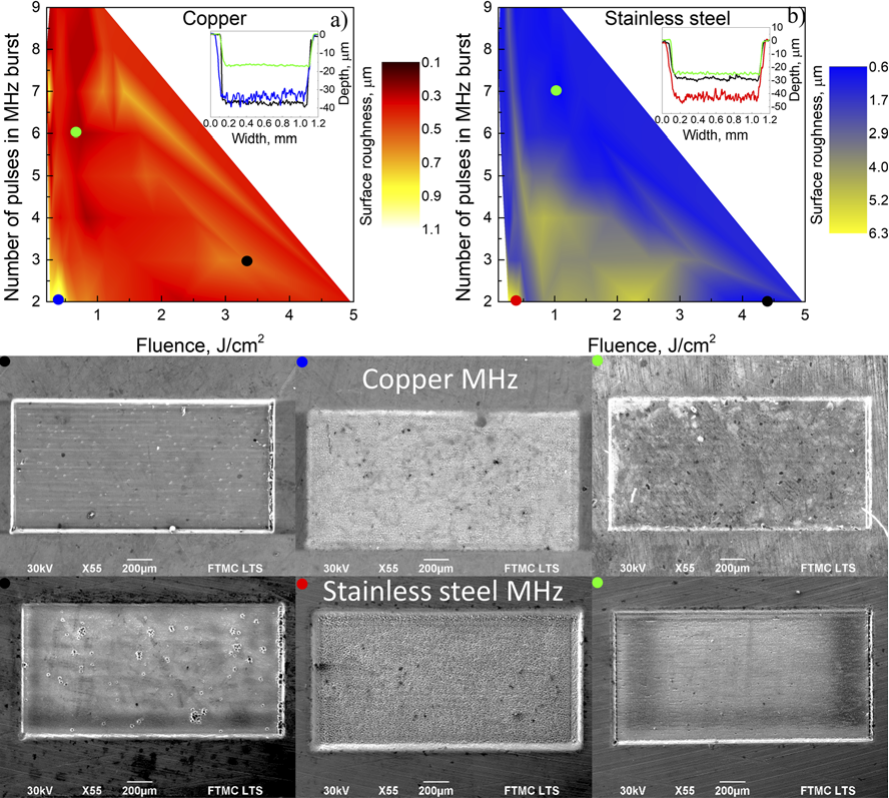
Surface roughness dependence on fluence and number of pulses in the MHz burst for (a) copper and (b) stainless steel. (a) For copper, black dot and solid line 3 pulses in the MHz burst, 3.3 J cm^−2^ fluence, 0.53 μm surface roughness; blue dot and solid line 2 pulses in the MHz burst, 0.4 J cm^−2^ fluence, 1.07 μm surface roughness; and green dot and solid line 6 pulses in the MHz burst, 0.8 J cm^−2^ fluence, 0.23 μm surface roughness. (b) For stainless steel, black dot and solid line 2 pulses in the MHz burst, 4.4 J cm^−2^ fluence, 0.63 μm surface roughness; red dot and solid line 2 pulses in the MHz burst, 0.6 J cm^−2^ fluence, 5.4 μm surface roughness; and green dot and solid line 7 pulses in the MHz burst, 1.1 J cm^−2^ fluence, 0.67 μm surface roughness. For all color dots, scanning electron microscope pictures are shown.

While using MHz burst mode with fluences lower than 0.5 J cm^−2^, the surface roughness was quite high, reaching around 1 μm in copper (Fig. 4a). We can also see scanning electron microscope pictures (SEM) in (Fig. 4). A rectangular cavity marked with a blue dot and two pulses in the MHz burst was used, and the depth is equal to 36 μm with a roughness of 1.07 μm. From 0.5 to 1 J cm^−2^ fluence, the surface roughness improved significantly in an interval from 0.2 μm to 0.5 μm. The lowest roughness has 4 or more pulses in the MHz burst. There are a few effects at play here. First, there is the incubation effect, which is that the ablation threshold decreases with an increasing number of pulses in the same spot.^25^ The second and most important factor is heat accumulation. Subsequent pulses reach the target material before the heat-affected zone has cooled off, so less energy is needed to remove the material.^26^ This is also evident when we compare the beam radii at the same fluences. With *N* = 2 and *N* = 3 pulses in the MHz burst, to achieve a fluence of approximately 0.7 J cm^−2^, the sample was moved 2.7 mm and 2.4 mm out of focus to increase the beam radii to 57.8 μm and 52.9 μm respectively. The beam radius was increased to only 37.4 μm for *N* = 4 and *N* = 5, to 31.8 μm for *N* = 6 to *N* = 8, and finally, to 24.8 μm for *N* = 9. The smaller beam radius allowed for greater heat accumulation in the area. As illustrated in Fig. 4, we can observe an SEM image with a significantly more even surface with a low roughness of 0.23 μm and an ablated depth of 18.1 μm. For fluences higher than 1 J cm^−2^, the roughness increased again to an interval of 0.5–0.7 μm. At higher fluences, particle shielding also has a stronger negative effect on scattering and absorbing incoming laser irradiation. We can see in Fig. 4 an SEM image marked with a black dot of a milled rectangle with a surface roughness of 0.53 μm. Žemaitis *et al.*^14^ showed that the highest ablation efficiency was reached when a MHz burst had 3 pulses. The aforementioned regime resulted in a relatively good quality of surface roughness compared to the other results. However, it could be improved by adding a polishing step using an *N* = 6 regime to decrease the surface roughness even further. Metals exposed to air are covered in an oxide layer with a thickness of up to a few micrometres. Incoming laser irradiation first interacts with the oxide, with a significantly higher ablation threshold^27^ and only afterwards with the metal itself. Žemaitis *et al.*^28^ demonstrated that a copper sample covered in a Cu_2_O oxide has three oxide transmission cases described by different *z/z*_R_ values, where *Z*_R_ = π*w*_0_^2^/(λ*M*^2^) *z*_R_ is the Rayleigh length, *z* is the sample position, *λ* is the wavelength of the laser irradiation, and *M*^2^ is the quality parameter of the Gaussian beam. The three transmission cases are as follows: when the sample is very out of focus and *z*/*z*_R_ ≫> ±1 linear absorption is dominant with a transmission value of *T*_0_ ≈ 60%, when the sample is not far from focus and *z*/*z*_R_ ≈ ±1 and is in the saturable absorption region with a transmission value of *T*_0_ ≈ 99%, and when the sample is in focal position with *z*/*z*_R_ ≈ 0, where nonlinear absorption exceeds absorption saturation in the oxide layer and transmission drops down to *T*_0_ ≈ 44%. However, although the difference in the transmission is quite high, its impact decreases significantly when multiple scans are performed on the target's surface.

We observe similar tendencies with stainless steel (Fig. 4b), but because stainless steel has a higher specific heat capacity 0.500 J (g^−1^ K^−1^)^29^ compared to copper 0.385 J (g^−1^ K^−1^), it takes more pulses and higher fluences to reach the optimal heat accumulation. For fluences lower than 0.8 J cm^−2^, the surface quality is quite poor. As depicted in (Fig. 4), visible graining can be observed in the SEM image with a red dot. The ablated depth is equal to 41.7 μm, and the surface roughness is 5.4 μm. For fluences higher than 0.8 J cm^−2^ and with 5 or more pulses in the MHz burst, we observed the optimal window for the highest surface quality on stainless steel. Similar to copper, the higher beam radii at the same 1.1 J cm^−2^ fluences in stainless-steel result in poorer surface quality. The surface roughness only reached a value lower than 1 μm value when the beam radius was decreased below 24.8 μm at *N* = 6 and a higher number of pulses. As demonstrated in (Fig. 4), an SEM image with a green dot showed a surface roughness of only 0.67 μm while using 7 pulses in the MHz burst. However, a similar finish quality can be achieved with fewer pulses in the burst but higher fluence, as can be observed in the SEM image with a black dot (Fig. 4), where 0.63 m surface roughness was achieved with a fluence of 4.4 J cm^−2^ and two burst pulses at MHz. Stainless steel had an overall higher surface roughness because the starting roughness was higher and equal to 0.5 μm compared to 0.1 μm of copper.

**Fig. 5:**
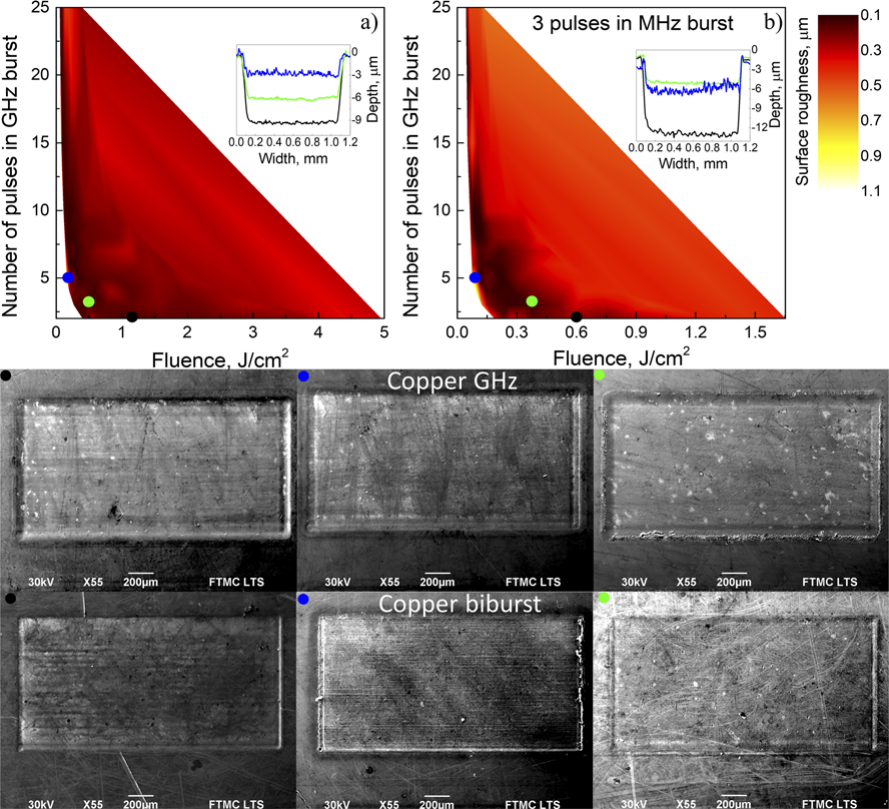
Surface roughness dependence on fluence and number of pulses in the (a) GHz burst and (b) biburst modes. (a) Black dot and solid line 2 pulses in the GHz burst, 1.1 J cm^−2^ fluence, 0.10 μm surface roughness; green dot and solid line 3 pulses in the GHz burst, 0.5 J cm^−2^ fluence, 0.10 μm surface roughness; and blue dot and solid line 5 pulses in the GHz burst, 0.16 J cm^−2^ fluence, 0.72 μm surface roughness. (b) *N* is the number of pulses in the MHz burst, and *P* is the number of pulses in the GHz burst. Black dot and solid line *N* = 3, *P* = 2 pulses in the biburst, 0.6 J cm^−2^ fluence, 0.20 μm surface roughness; blue dot and solid line *N* = 3, *P* = 5 pulses in the biburst, 0.07 J cm^−2^ fluence, 0.74 μm surface roughness; and green dot and solid line *N* = 3, *P* = 3 pulses in the biburst, 0.4 J cm^−2^ fluence, 0.08 μm surface roughness. For all color dots, scanning electron microscope pictures are shown.

Using the GHz laser polishing regime, the surface roughness of copper was in the range of 0.1–0.35 μm for the vast majority of fluence and number of pulses within the burst combinations. SEM images of 0.1 μm roughness are shown for two and three pulses in the GHz burst, as illustrated in Fig. 5, and marked with black and blue dots, respectively, with different fluences in which the first is 0.5 J cm^−2^ and the other 1.1 J cm^−2^. Even with the worst outcomes for the GHz burst polishing regime, the roughness was only 0.53 μm, as can be observed in the SEM image in Fig. 5, and has a blue dot. This was the worst combination of parameters with 5 pulses at 0.22 J cm^−2^ fluence when the sample was 3.8 mm out of the focal plane. GHz burst mode uses heat accumulation to its fullest effect because of the incredibly small time window between subsequent pulses. However, note that it has been shown that the ablation efficiency drops by about 90%^30^ in the GHz burst mode, so it should only be used as a polishing step. As shown in Fig. 5a, the polishing process is very parameter independent.

In the biburst mode polishing, the surface roughness again becomes more parameter dependent. All the results for the biburst combinations are presented in the ESI.† The vast majority of parameter combinations lead to a surface roughness lower than 0.5 μm. However, using 10 or fewer pulses in the GHz burst and fluences for each sub-pulse lower than 0.6 J cm^−2^ result in roughness lower than 0.2 μm. SEM images are shown in (Fig. 5b). High-quality polishing was shown in SEM images with black and green dots, corresponding to *N* = 3, *P* = 2, 0.10 μm roughness and *N* = 3, *P* = 3, 0.08 μm roughness, respectively. Again, the low surface quality is observed only in the very out of focus 3.1 mm and more, milled cavities, and low fluences, such as in SEM image with a blue dot (Fig. 5b). Overall, bibursts result in similar surface roughness at optimal parameters but slightly higher roughness for sub-optimal ones. These tendencies remain unchanged depending on the number of pulses in the MHz burst, as demonstrated in the ESI.†

**Fig. 6:**
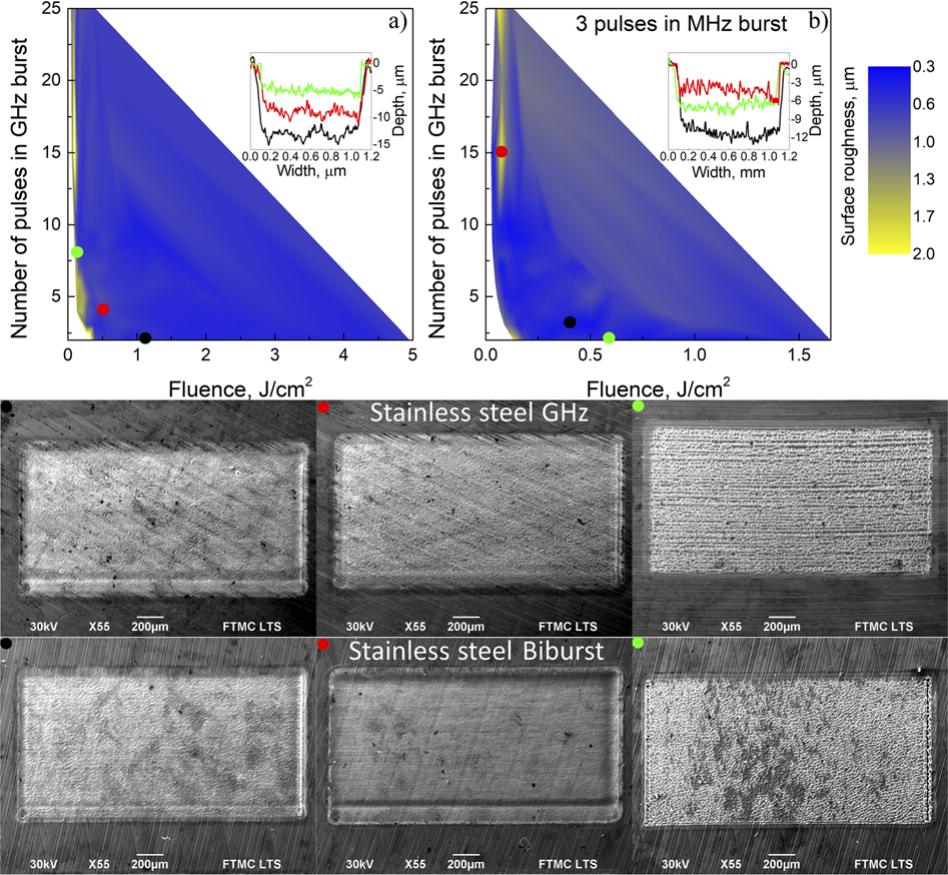
Surface roughness dependence on fluence and number of pulses in the (a) GHz burst and (b) biburst modes. (a) Black dot and solid line 2 pulses in the GHz burst, 1.1 J cm^−2^ fluence, 0.66 μm surface roughness; red dot and solid line 3 pulses in the GHz burst, 0.5 J cm^−2^ fluence, 0.55 μm surface roughness; and green dot and solid line 8 pulses in the GHz burst, 0.1 J cm^−2^ fluence, 1.4 μm surface roughness. (b) *N* is the number of pulses in the MHz burst, and *P* is the number of pulses in the GHz burst. Green dot and solid line *N* = 3, *P* = 2 pulses in the biburst, 0.6 J cm^−2^ fluence, 0.57 μm surface roughness; black dot and solid line *N* = 3, *P* = 3 pulses in the biburst, 0.4 J cm^−2^ fluence, 0.45 μm surface roughness; and red dot and solid line *N* = 3, *P* = 15 pulses in the biburst, 0.05 J cm^−2^ fluence, 1.89 μm surface roughness. For all color dots, scanning electron microscope pictures are shown.

As with copper, the GHz burst results in the highest quality on stainless steel with vastly different parameters. As illustrated in Fig. 6a, SEM images with red and black dots with a surface roughness of 0.66 μm and 0.55 μm were reached with 2 pulses in the GHz burst, 1.1 J cm^−2^ fluence, and 3 pulses in the GHz burst, 0.5 J cm^−2^ fluence. With copper, poor-quality results are received only when the sample is 3.8 mm or more out of focus when the fluence is 0.1 J cm^−2^. An example is shown with an SEM image with a green dot, with a corresponding surface roughness of 1.4 μm.

All the biburst results on stainless steel are shown in the ESI.† Surface roughness (Fig. 6b) was similar to the GHz burst mode at optimal parameters with a roughness of 0.57 μm at *N* = 3, *P* = 2 in the SEM image with a black dot and 0.45 μm at *N* = 3, *P* = 2 SEM image with a red dot. High surface roughness is also shown in the SEM image with a green dot, corresponding to a surface roughness of 1.89 μm at *N* = 3, *P* = 15.

## Conclusions

Using 210 fs laser pulses in the burst and biburst regimes, copper and stainless-steel samples were ablated to receive the highest surface quality. It is shown that both MHz and GHz bursts are useful in reducing surface roughness, with MHz burst having the advantage of higher ablation efficiency, while GHz bursts were very parameter independent for the surface quality. The GHz bursts use heat accumulation better because of the shorter time between pulses. *R*_a_ surface roughness of 0.23 μm, 0.1 μm and 0.08 μm were achieved for copper in the MHz, GHz and biburst modes, respectively. Additionally, many parameters yielded <0.15 μm *R*_a_ for the GHz burst mode. The same tendencies were seen for stainless-steel. The lowest surface roughness for the MHz burst was 0.63 μm, 0.55 μm for the GHz burst, and 0.45 μm for the biburst mode. Using the MHz burst mode, the ablation efficiency is the highest while using 3 pulses in the bursts at the optimal fluence, but the surface roughness is lowest with 6 pulses within the burst. GHz and biburst modes have a significantly lower ablation efficiency but a significantly higher polishing quality that could be used as a finishing step to achieve the desired surface roughness.

## Author contributions

M. Gaidys wrote the manuscript, did all the stylus profiler measurements and SEM imaging, performed beam measurement experiments, A. Žemaitis performed polishing experiments, all authors read and corrected the manuscript.

## Conflicts of interest

There are no conflicts to declare.

## Supplementary Material

RA-013-D2RA05208C-s001
