# Supplementary material for: Efficient surface polishing using burst and biburst mode ultrafast laser irradiation
Source: RSC Adv. 2023 Jan 25;13(6):3586–91. doi: 10.1039/d2ra05208c (PMC9890557; doi:10.1039/d2ra05208c)
Supplement: RA-013-D2RA05208C-s001 [file RA-013-D2RA05208C-s001.pdf]

Electronic Supplementary Material (ESI) for RSC Advances.
This journal is © The Royal Society of Chemistry 2023

Supplementary Material

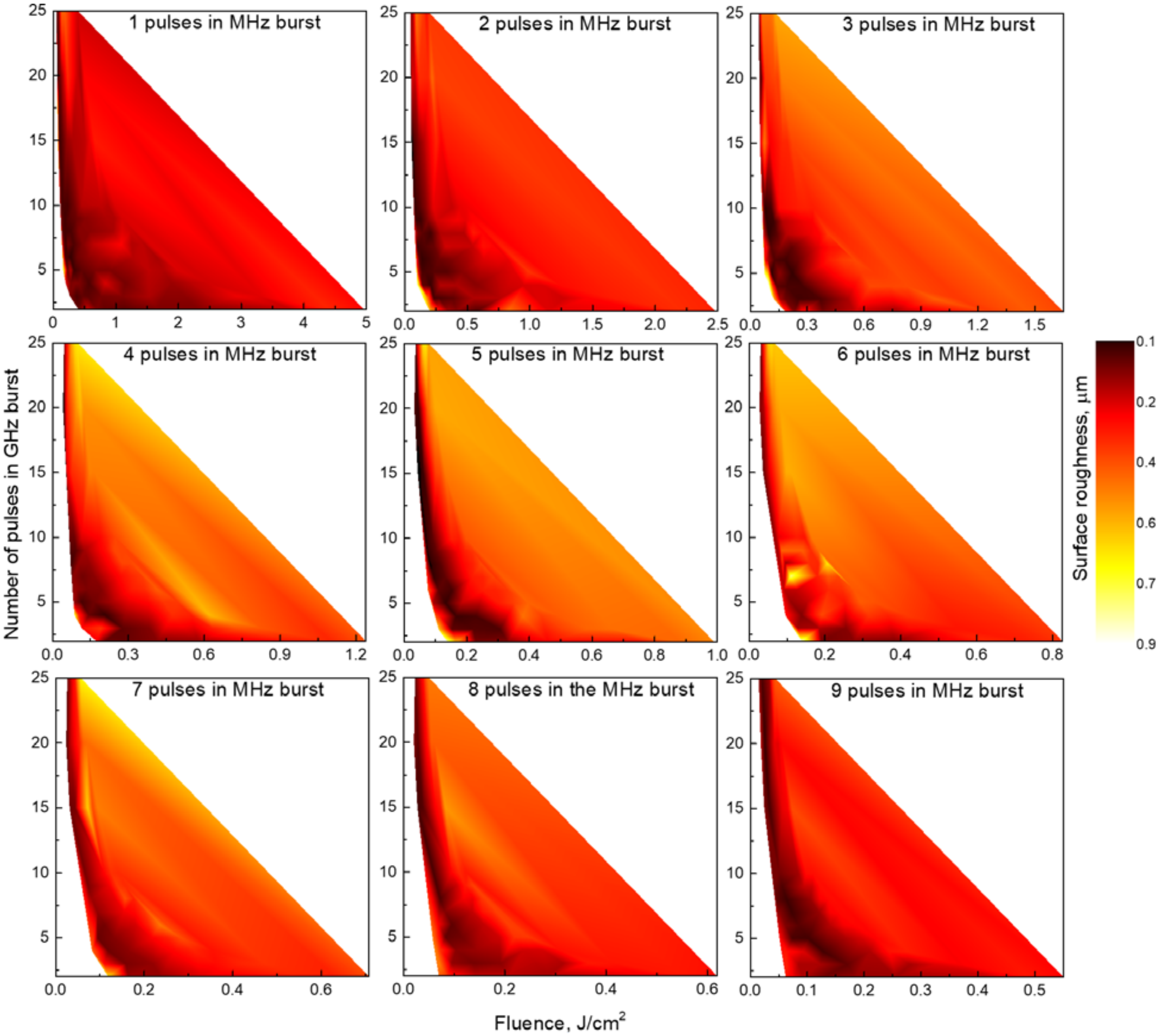

Fig 7. Surface roughness dependence on fluence and number of pulses in the biburst on copper.

In (Fig. 7) 1 pulse in the MHz burst with changing numbers of pulses in the GHz burst is the GHz burst regime. 2 to 9 pulses in the MHz burst with changing numbers of pulses in the GHz burst is the biburst regime. In copper the biburst regime resulted in slightly higher surface roughness on average compared to the GHz regime. The best results were comparable with 0.1 um for the GHz burst regime and 0.08 um for the biburst mode, respectively. However, the biburst mode was significantly more parameter dependent. The average roughness for most of the biburst regimes was in the 0.4 – 0.6 um interval. The incubation effect saturates with such an increased amount of pulses within the bursts and increasing the number of pulses further does not increase the surface quality compared to the GHz burst mode.

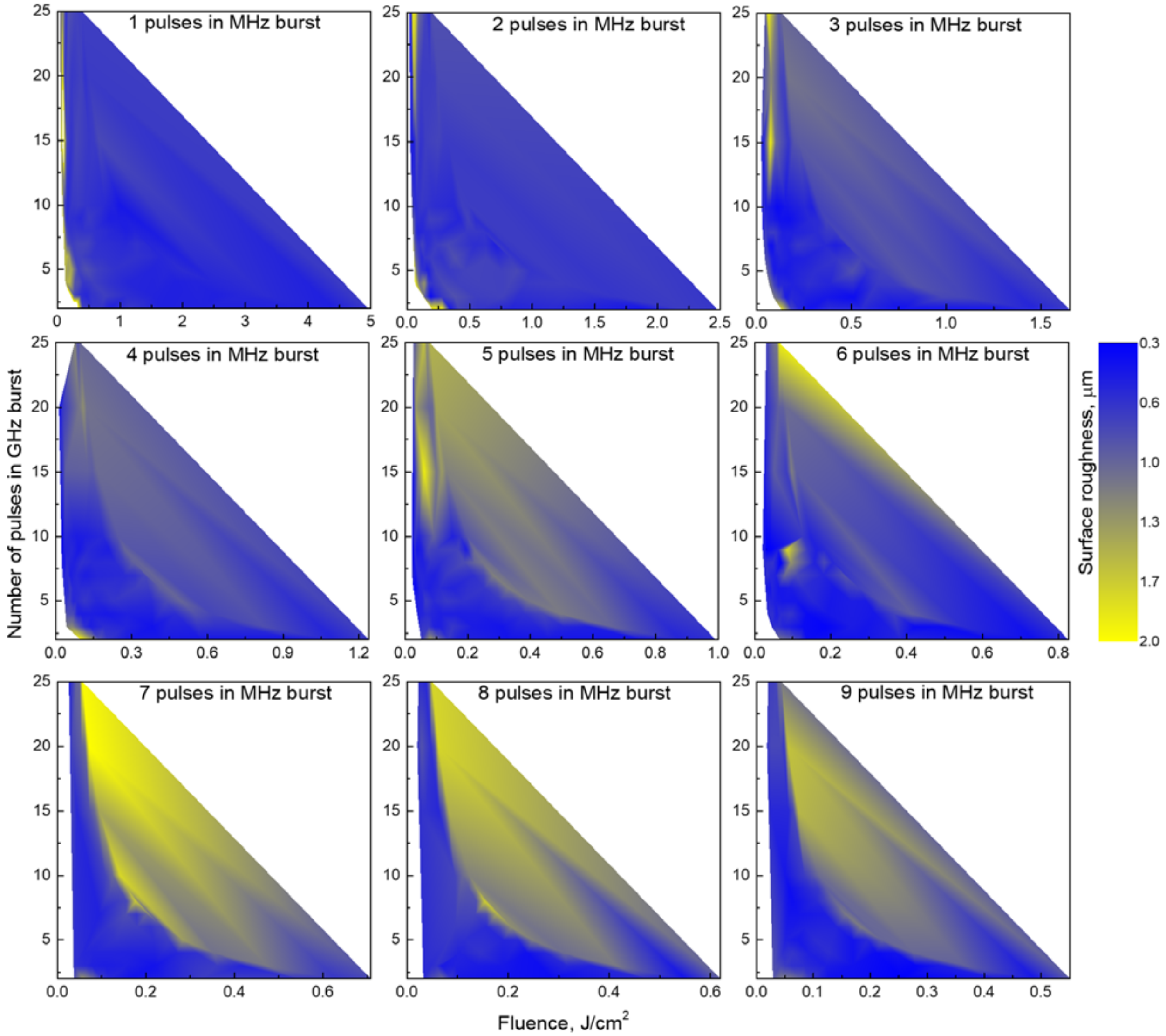

Fig. 8. Surface roughness dependence on fluence and number of pulses in the biburst on stainless steel.

For stainless-steel (Fig. 8) similar results are visible. GHz burst mode is the best at reducing the surface roughness and the average roughness for biburst mode is in the 0.6 – 1.3 um interval. The roughness was higher compared to copper due to the higher initial surface roughness of the samples.
